# Prevalence and Associated Risk Factors of Cognitive Frailty: A Systematic Review and Meta-Analysis

**DOI:** 10.3389/fnagi.2021.755926

**Published:** 2022-01-28

**Authors:** Tao Zhang, Yan Ren, Ping Shen, Shixie Jiang, Yanrong Yang, Yan Wang, Zheng Li, Ying Yang

**Affiliations:** ^1^School of Medicine, Zunyi Medical University, Zunyi, China; ^2^Sichuan Provincial People's Hospital, Sichuan Academy of Medical Sciences, Chengdu, China; ^3^Department of Geriatrics, Fifth People's Hospital of Chengdu, Chengdu, China; ^4^College of Life and Science, Chengdu University of Traditional Chinese Medicine, Chengdu, China; ^5^Department of Psychiatry and Behavioral Neurosciences, University of South Florida, Tampa, FL, United States; ^6^Department of Neurology, Fifth People's Hospital of Chengdu, Chengdu, China; ^7^School of Computer Science and Technology, Chongqing University of Posts and Telecommunications, Chongqing, China

**Keywords:** cognitive frailty (CF), associated factor, prevalence, frailty, cognitive

## Abstract

**Objective:**

Currently, the prevalence of CF (Cognitive Frailty) is not very clear, and the relationship between CF and its associated risk factors has not been accurately evaluated. Therefore, it is necessary to conduct a systematic review and meta-analysis further to understand CF's prevalence and associated factors.

**Methods:**

Embase, PubMed, Web of Science, Ovid, and Cochrane were systematically searched for articles exploring the prevalence of CF, the deadline of searching date was up to March 2021. For the prevalence of CF, the events of CF and the total number of patients in every included study were extracted to estimate the prevalence of CF. For associated factors of CF, Odds Ratios (ORs) with (corresponding) 95% confidence intervals (CIs) were used for estimations.

**Results:**

Firstly, the estimated prevalence of CF I (Cognitive Frailty in the model I) was 16%, 95% CI (0.13–0.19), and the estimated prevalence of CF II (Cognitive Frailty in model II) was 6%, 95% CI (0.05–0.07). Secondly, both lower engagement in activities and age were calculated to be independent risk factors of CF, and the OR (95% CI) was 3.31 (2.28–4.81) and 1.10 (1.04–1.16), respectively. Finally, depression was also a prominent risk factor of CF, with the overall OR (95% CI) as 1.57 (1.32–1.87).

**Conclusion:**

CF was a high prevalence in community older. The various assessment scales and the different cutoff values of diagnostic criteria would affect the prevalence of CF. Lower engagement in activities, age, and depression was the risky factor of CF.

**Systematic Review Registration:**

http://www.crd.york.ac.uk/PROSPERO/, identifier: CRD42019121369.

## Introduction

Frailty, a critical intermediate status of the aging process and a reversible condition, is a multidimensional clinical syndrome that includes physical, cognitive, social, and psychological dimensions or phenotypes (Clegg et al., 2013; Rodríguez-Mañas et al., 2013; Sugimoto et al., 2018). Numerous studies have confirmed that cognitive impairment is significantly associated with physical frailty, as both cognitive impairment and physical frailty often co-occur in older people (Avila-Funes et al., 2009; Boyle et al., 2010; Auyeung et al., 2011; Malmstrom and Morley, 2013; Shimada et al., 2013). Based on previous research, the consensus from the International Academy on Nutrition and Aging and the International Association of Gerontology and Geriatrics (IANA-IAGG) proposed the operational definition of cognitive frailty (CF) as the co-existence of physical frailty and mild cognitive impairment (MCI) in the absence of dementia (Kelaiditi et al., 2013). Individuals with CF carry a higher risk of developing dementia and mortality in comparison to healthy older adults (Solfrizzi et al., 2017a), as well as a higher risk than older adults with either physical frailty or cognitive impairment alone (Avila-Funes et al., 2009; Feng et al., 2017; Shimada et al., 2018; Zhang et al., 2021). Notably, CF is a potentially reversible condition, unlike dementia (Clegg et al., 2013). It is a state of reduced cognitive reserve, occurring at an intermediate stage between age-related cognitive changes and neurodegenerative diseases (Dorner et al., 2013; Morley et al., 2013). However, CF by itself may still lead to the following adverse outcomes: decline in physiological function, disability, hospitalization, and dementia (Avila-Funes et al., 2009; Solfrizzi et al., 2017a,b; Shimada et al., 2018). Therefore, CF preventive and health promotion strategies need to be implemented in the early stages or the reversible stage.

Owing to different assessment measures and diagnostic criteria, the prevalence of CF varies significantly among studies. There is currently no gold standard for diagnosing CF and no evident estimated prevalence of CF in community-dwelling individuals.

Moreover, in recognition of the importance of CF and the perniciousness of adverse health, a significant number of studies have focused on the risk factors for it. However, the main associated factors of CF have also varied in different studies, and results have been controversial. For example, Katayama et al. (2021) reported that sex was independently associated with CF. However, Chu et al. (2019) reported that sex did not show a significant association with CF. Furthermore, Xie et al. (2021) reported that depression was independently associated with CF, but Chu et al. (2019) reported that depression was not. Therefore, it is crucial to objectively evaluate the risk factors of CF with more rigorous scientific methods.

Given those as mentioned earlier, we have conducted a systematic review and meta-analysis to clarify CF's prevalence and associated risk factors in community-dwelling older adults.

## Methods

### Inclusion Criteria

1) We defined CF as the co-existence of frailty and cognitive impairment, so included studies must evaluate cognition level and frailty.
2) The original study must include the number of individuals with CF and the total population size.
3) The included population is community-dwelling.
4) Multiple papers were generated from the same data set; only the most relevant study and the larger sample were included.

### Exclusion Criteria

1) The original study did not involve or could not calculate the number of those diagnosed with CF.
2) Data cannot be obtained, even after contacting the corresponding author of a study.
3) Literature reviews, case reports, animal studies, or conference abstracts.
4) Non-English studies.

### Data Sources and Search Strategy

**Figure 1:**
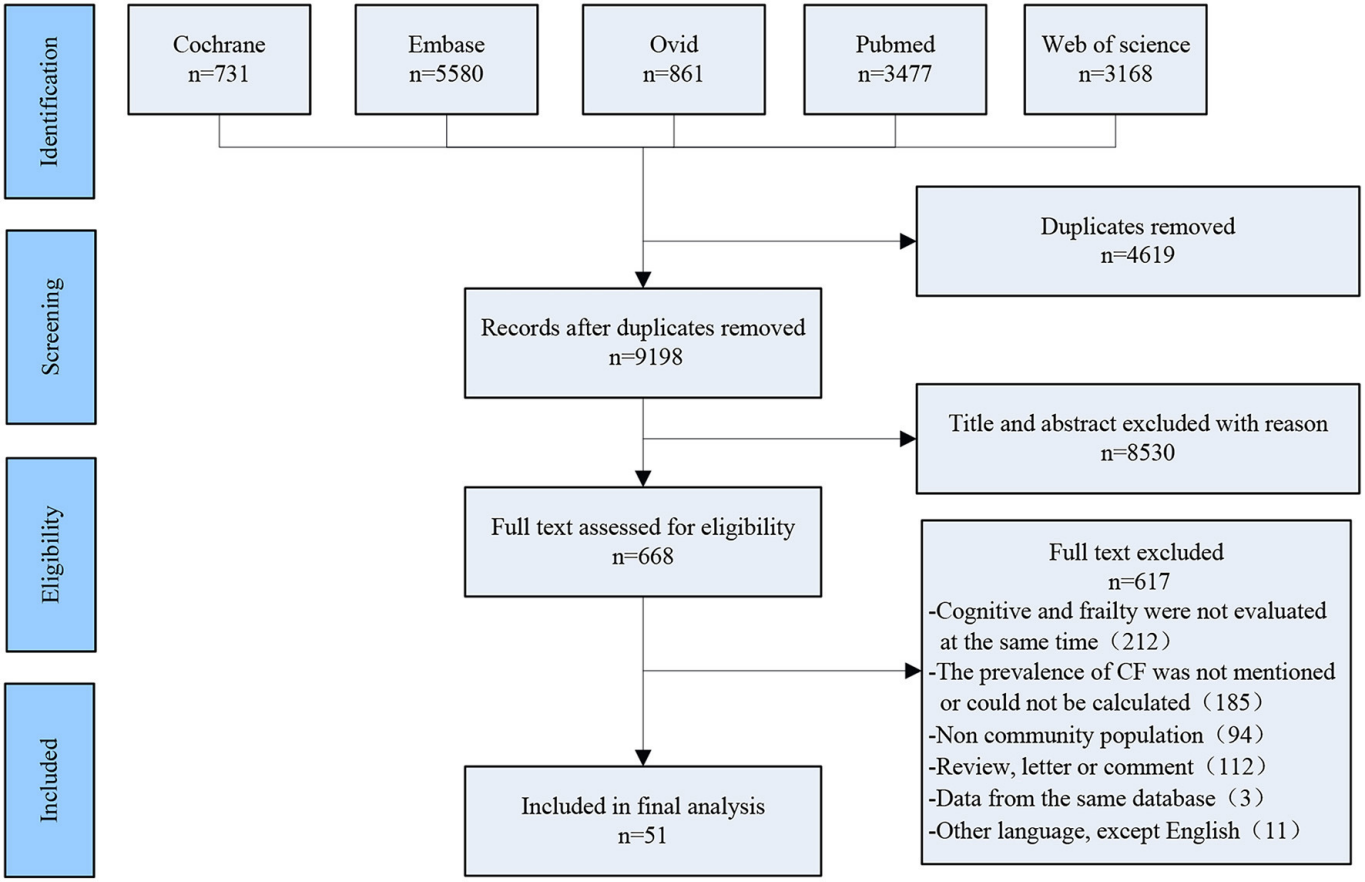
The PRISMA diagram of the study selection.

Two researchers (Tao Zhang and Yan Ren) independently searched the following electronic databases: Cochrane, PubMed, Web of Science, Ovid, and EMBASE, the deadline of searching date was up to March 2021. Search terms were as follows: [(frailty [Mesh Terms]) OR (frail^*^[Title/Abstract])] AND (cogniti^*^[Title/Abstract]). After removing duplicates, 9,198 articles were screened, and the screening process of included studies is shown in Figure 1.

### Data Extraction and Study Selection

Firstly, two reviewers (Tao Zhang and Yan Ren) screened the titles and abstracts from searches and independently selected relevant studies, which met the inclusion criteria. Secondly, Tao Zhang and Yan Ren decided on the studies for final inclusion after reviewing the full text of potential studies. Thirdly, once the quantitative data in the original study met the inclusion criteria, we extracted the number of events of CF and other important information. Fourth, we also collected the adjusted OR and 95% (CI), which evaluated the associated risk factors of CF. Lastly, any disagreement in selection was referred to the arbitrator (Ying Yang). We also contacted the corresponding author of the original studies for additional information if required.

### Risk of Bias (Quality) Assessment

Two reviewers (Ping Shen and Yan Ren) independently assessed the risk of bias of the included studies by using a tool explicitly designed for assessing the risk of bias of prevalence studies (Hoy et al., 2012) (The full details of this tool are presented in Supplementary Table 4). In brief, it involves a total of 10 items that contain three domains: measurement bias, selection bias, and analysis bias. The answer to each item was, “Yes (low risk),” or “No (high risk).” When ≥ 8 items answered, “Yes (low risk),” low risk of bias was considered, a moderate risk of bias when 6 to 7 items were answered as “YES (low risk)”; and a high risk of bias when ≤ 5 items were answered as “YES (low risk).” Any disagreement among the reviewers was discussed with the arbitrator (Ying Yang).

### Strategy for Data Synthesis

Firstly, the actual events of CF and the total number of patients in every included study were extracted to estimate the prevalence of CF. Secondly, we collected the adjusted OR (95% CI), which evaluated the associated risk factors of CF. Finally, we adopted a random-effects model if the heterogeneity test significantly detected statistical difference (I^2^ > 50%) or otherwise used a fixed-effects model. All analyses were performed using STATA software (version 16.0, STATA Corp., College Station, TX, USA).

## Results

### Included Studies and Demographics

A total of 51 studies and 123,771 patients were included in our analysis (Supplementary Table 2), and 64,784 were observed to be female. All the patients were recruited from the community. Thirty-three among the included 51 studies were pooled by meta-analysis. Patients were included from different countries, including France, Mexico, Australia, Netherlands, Spain, Italy, England, Malaysia, Singapore, India, Thailand, Japan, Korea, China, Brazil, Canada, and the United States. Among the included studies, 20 were considered moderate quality, which involves a moderate risk of bias, and 31 studies were considered high quality, with a lower risk of bias.

### Meta-Analysis Results

#### Prevalence of Cognitive Frailty

**Figure 2:**
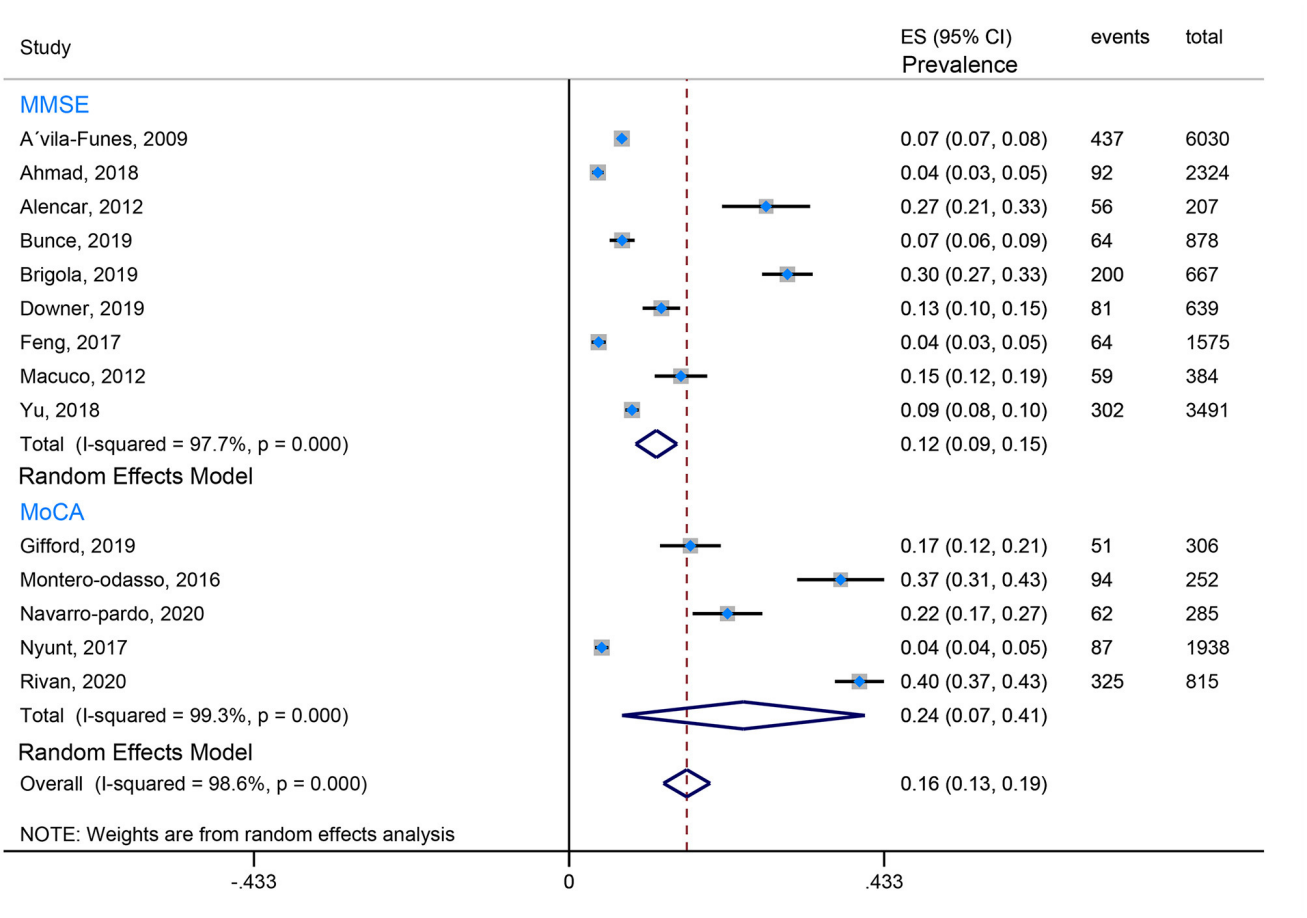
The forest plot pooled the prevalence of CF I.

**Figure 3:**
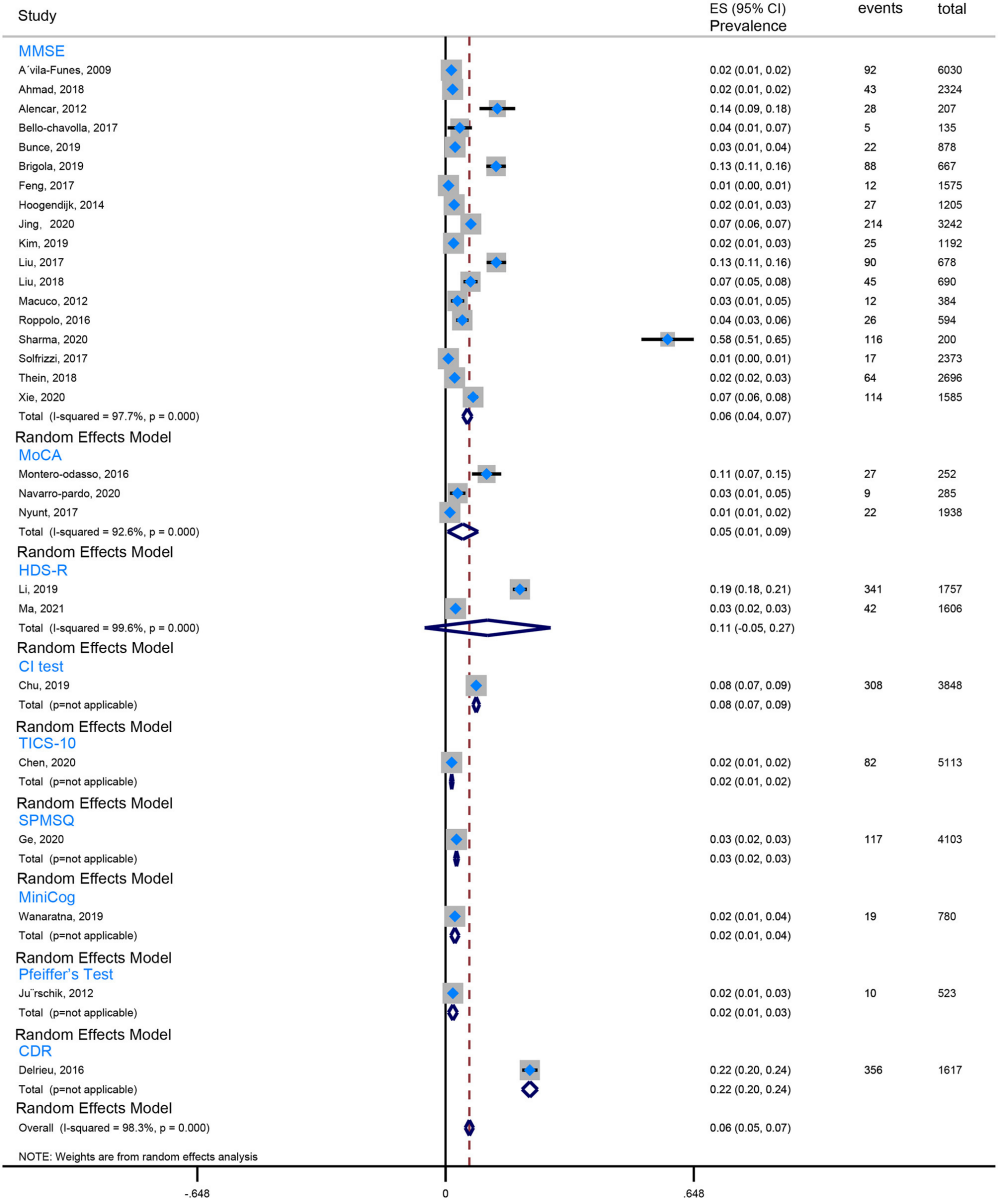
The forest plot pooled the prevalence of CF II.

Our review found that the Fried criteria were most commonly used to define frailty in community residents. A total of 35 studies reported the prevalence of Fried-defined frailty. Otherwise, 5 included studies applied the FRAIL scale to define frailty, and 3 included studies applied the FI (Frailty index). The remaining evaluation tools were not very common or standardized assessment tools. Moreover, the assessment tools of cognitive function are also observed to be varied. Twenty-seven studies used the MMSE (Mini-Mental State Examination) to evaluate cognition, 5 studies utilized the MoCA (Montreal Cognitive Assessment), 3 studies took advantage of the NCGG-FAT (National Center for Geriatrics and Gerontology-Functional Assessment Tool) to assess cognitive function, 2 studies applied the CDR (Clinical Dementia Rating Scale), and 2 studies used the HDS-R (Revised Hasegawa's Dementia Scale). In this case, the prevalence of CF is quite different because of the ununified assessment scale and various cutoff values, which from the lowest prevalence 0.71% (Solfrizzi et al., 2017b) to the highest prevalence 58% (Sharma et al., 2020). To minimize the heterogeneity, we combined data using the Fried-defined frailty; however, given different cutoff values, further categorization was conducted; therefore, we divided them into CF I (cutoff value ≥ 1 in Fried Criteria Scale) and CF II (cutoff value ≥ 3 in Fried Criteria Scale) to conduct our meta-analysis. We found that the estimated prevalence of CF I was 16%, 95% CI (0.13–0.19) (Figure 2), and the estimated prevalence of CF II was 6%, 95% CI (0.05–0.07) (Figure 3). There are three reasons why we divided into CF Model I and CF Model II. Firstly, CF was composed of cognitive impairment (MCI) and frailty. CF Model I included the whole population classified as MCI with frailty, and CF Model I emphasized the overall prevalence in the globally high risky population. Secondly, the CF Model II represents a more strict cutoff value, and CF Model II has excluded someone diagnosed with pre-frailty. Therefore, CF Model II could emphasize the severity of CF. Thirdly, internal inconsistency is more evident in terms of validity and credibility if we consider model III (cutoff value from 1 to 3 in Fried Criteria Scale) (Hao et al., 2018; Sharma et al., 2020). Thereby, we adopt CF Model I and CF Model II to assess the prevalence of CF.

### Associated Risk Factors of Cognitive Frailty

#### Age

**Figure 4:**
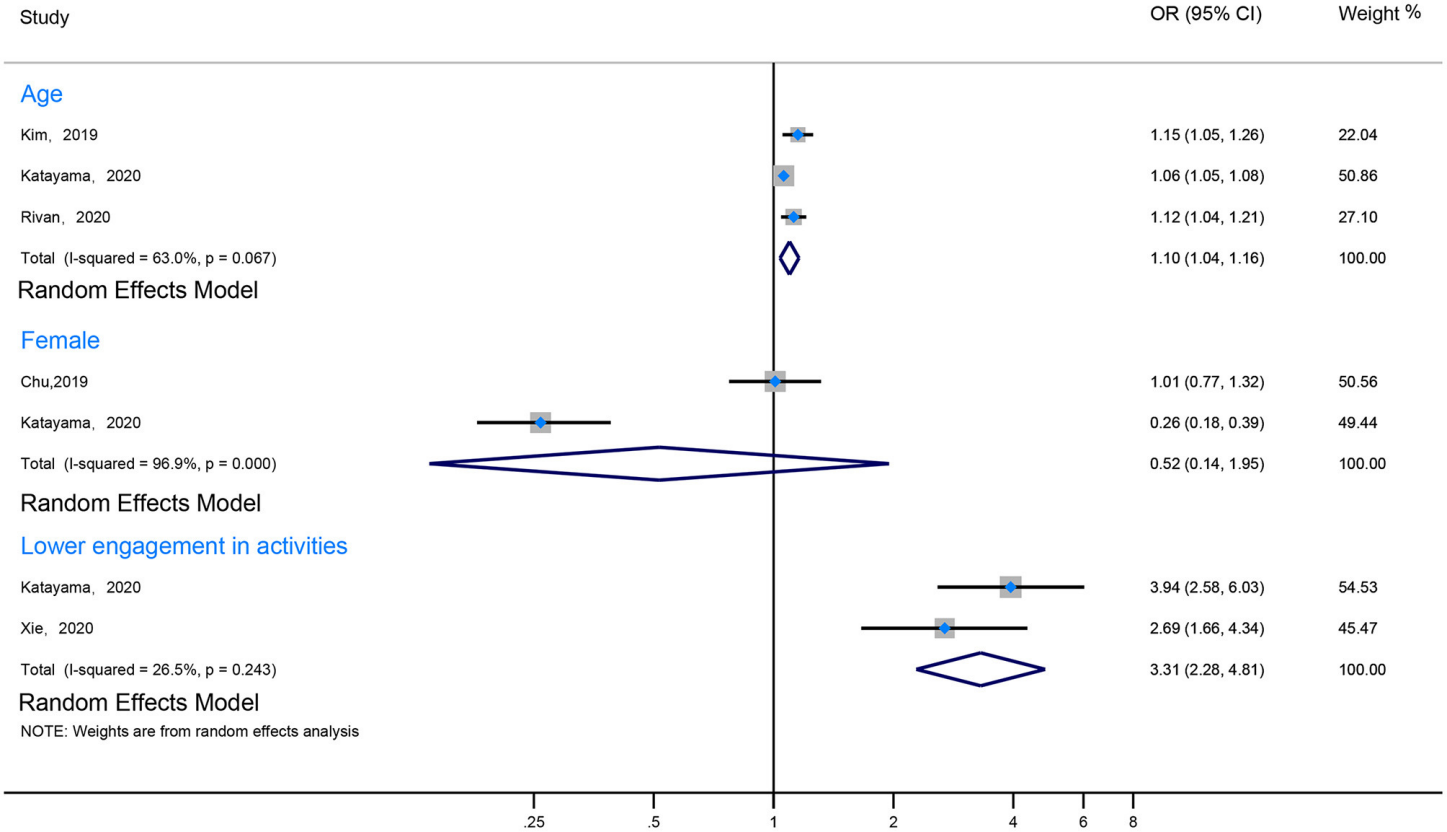
The forest plot illustrated the associated risk factors about CF.

Eight studies revealed that the prevalence of CF increased with age (Ma et al., 2017; Chu et al., 2019; Kim et al., 2019; Navarro-Pardo et al., 2020; Rivan et al., 2020; Ruan et al., 2020; Katayama et al., 2021; Xie et al., 2021). Among them, three studies mentioned that the prevalence of CF in elderly individuals over 80 years of age was significantly higher than in younger groups (Navarro-Pardo et al., 2020; Ruan et al., 2020; Xie et al., 2021). Notably, Ruan et al. (2020) reported that individuals aged 80 years were at a higher risk than those aged 60–69 years (OR 19.71, 95% CI 13.49–28.79). Moreover, 3 other studies reported that age per 1-year increment was an associated risk factor of CF (the pool data: I^2^ 63%, OR 1.10, 95% CI 1.04–1.16) (Kim et al., 2019; Rivan et al., 2020; Katayama et al., 2021). However, in one study, Navarro-Pardo found that in comparison with those aged 60–64 years, age was not a risk factor for CF in those younger than 80 years of age (Figure 4; Supplementary Table 3).

#### Gender

Two of the included studies reported an association between gender and CF (Chu et al., 2019; Ruan et al., 2020). One of them reported that gender was an independent risk factor of CF (Chu et al., 2019). However, the pooled data indicated that gender was not found to be an independent risk factor for CF (I^2^ 96.9%, OR 0.52, 95% CI 0.14–1.95) (Figure 4; Supplementary Table 3).

#### Physical Activity

Because the quantitative index of activity varied significantly between studies, we divided them into two groups: the more active group and the less active group, according to the description of the study. Two included studies addressed the relationship between activities and CF (I^2^ 26.5%, OR 3.31, 95% CI 2.28–4.81) (Katayama et al., 2021; Xie et al., 2021). One of the studies (Katayama et al., 2021) reported that different types of activities (such as going-out activities, cognitive and physical activities, and multidomain activities) had different effects on the prevalence of CF (OR 1.76, 95% CI 1.47–2.11; OR 2.80, 95% CI 1.97–3.97; OR 3.94, 95% CI 2.58–6.03; respectively) (Figure 4; Supplementary Table 3).

#### Negative Emotional State

**Figure 5:**
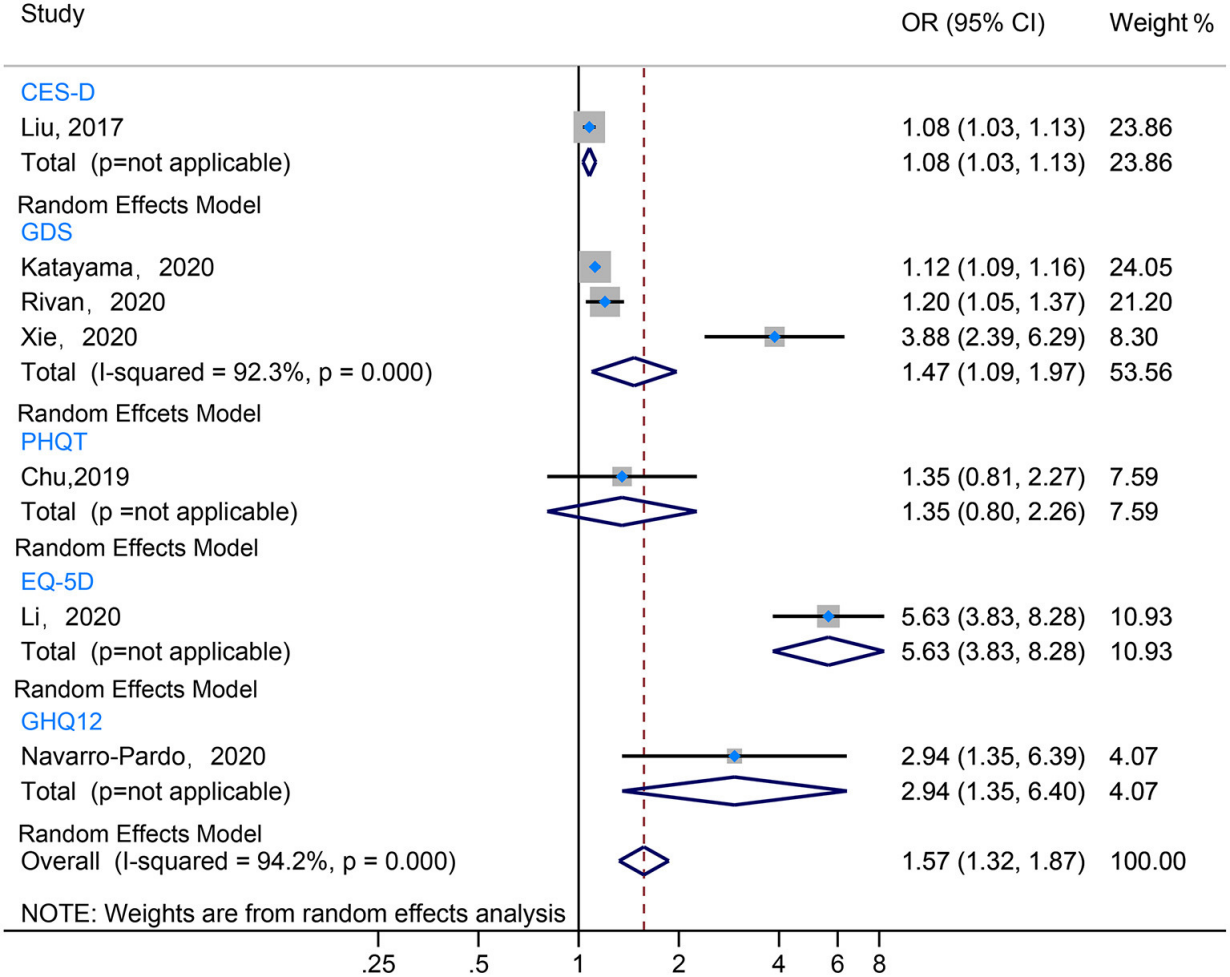
The forest plot illustrated the OR for various depression assessment scales estimating risk factor of CF.

Results are presented in Figure 5 and Supplementary Table 3. The meta-analysis of the 7 studies included suggested that negative emotion was associated with a statistically significant increased risk of the prevalence of CF (I^2^ = 94.2%, OR = 1.57, 95% CI 1.32–1.87) (Liu et al., 2018; Chu et al., 2019; Li et al., 2020; Navarro-Pardo et al., 2020; Rivan et al., 2020; Katayama et al., 2021; Xie et al., 2021). Of them, five studies revealed that patients with depression had a higher prevalence of CF (Liu et al., 2018; Navarro-Pardo et al., 2020; Rivan et al., 2020; Katayama et al., 2021; Xie et al., 2021). The pooled data showed that depression assessed by GDS was an independent risk factor of CF (I^2^ = 92.3%, OR = 1.47, 95% CI 1.09–1.97). However, Chu et al. (2019) reported that depression assessed by PHQT was not a risk factor for CF prevalence. In addition, depression with anxiety also was found to have a higher prevalence of CF (Li et al., 2020).

#### Education

Four included studies addressed the relationship between education level and CF (Chu et al., 2019; Navarro-Pardo et al., 2020; Ruan et al., 2020; Katayama et al., 2021). Due to severe heterogeneity among standards for evaluating education levels, a meta-analysis could not be performed. One included study (Chu et al., 2019) revealed that the incidence of CF was lower in those with a high school degree and higher in those with <8 years of education. However, education for more than 1–4 years did not significantly reduce the prevalence of CF. Nevertheless, another included study found that people with 6–12 years of education have a lower prevalence of CF than those under 6 years of education (Ruan et al., 2020). Navarro-Pardo et al. (2020) reported that those with lower years of education had an increased risk of CF compared with those with more than 7 years. When treated as continuous data, education per 1-point increment was positively associated with CF (OR 0.95, 95% CI 0.91–0.99) (Katayama et al., 2021) (Supplementary Table 3). Further original studies may focus on the relationship between education level and risk of CF, especially the dose-response relationship between CF and continuous data of education level.

#### Marital Status

One included study (Ruan et al., 2020) suggested that marital status, whether married (OR 0.995, 95% CI 0.327–3.025) or widowed (OR 1.802, 95% CI 0.564–5.757), possessed no correlation with the prevalence of CF compared with those who are single (Supplementary Table 3).

#### Social Participation and Sleep Problems

Xie et al. (2021) proposed that more social participation was a protective factor for CF (OR 0.61, 95% CI 0.39–0.96). The study also found that insomnia was a risk factor for CF. Moreover, daily insomnia was observed to be more harmful than occasional insomnia (OR 2.38, 95% CI 1.33–4.26; OR 1.84, 95% CI 1.07–3.17; respectively) (Supplementary Table 3).

#### Nutrition

Four of the included studies reported an association between nutrition and CF (Liu et al., 2018; Kim et al., 2019; Rivan et al., 2020; Katayama et al., 2021). One study (Liu et al., 2018) defined malnutrition according to the Mini-Nutritional Assessment (MNA), another one (Kim et al., 2019) evaluated nutrition by the CNAQ (Council on Nutrition Appetite Questionnaire). Finally, Katayama et al. (2021) used skeletal muscle mass index (ASM) to correlate nutritional status indirectly. These assessment tools indicated that the lower the nutritional status, the higher the prevalence of CF (OR 0.869, 95% CI 0.766–0.986; OR 0.736, 95% CI 0.628–0.863; OR 0.82, 95% CI 0.78–0.87; respectively). There are also different anthropometric results (Kim et al., 2019; Katayama et al., 2021) (calf circumference, total body fat, and body mass index) and biochemical (Rivan et al., 2020; Katayama et al., 2021) indicators (albumin and vitamin D) that may be used to reflect the nutritional status. Individuals who had a thinner calf circumference, higher total body fat, lower albumin, and lower vitamin D showed an increased CF prevalence (OR 0.748, 95% CI 0.625–0.895; OR 1.04, 95% CI 1.01–1.07; OR 0.45, 95% CI 0.34–0.59; OR 0.362, 95% CI 0.141–0.930; respectively). Body mass index was not a risk factor associated with CF (Supplementary Table 3).

### Sensitivity Analysis

**Figure 6:**
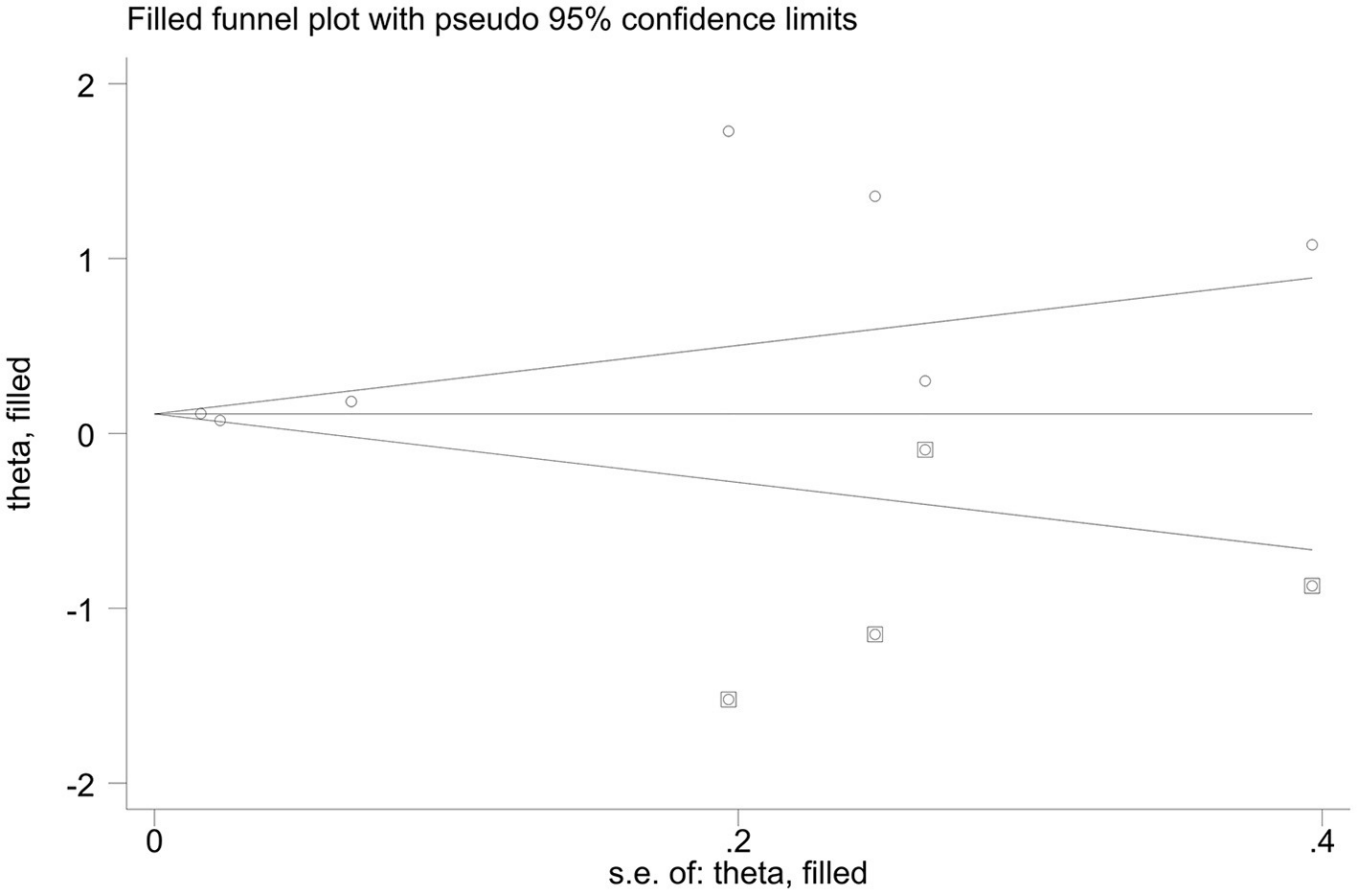
Funnel plots of non-parametric trim-and-fill method regards the OR for various depression assessment scales estimating risk factor of CF.

When calculating the prevalence of CF, a Begg's test and an Egger's test were employed, indicating some evidence for publication bias. However, we used the command to test the robustness of our results. By excluding one study at a time, our results were robust. Begg's test showed no publication bias regarding the OR for subgroup analysis of various depression assessment scales. However, Egger's test (*p*-value for Egger's test = 0.045) indicated some evidence for publication bias. Next, we performed a non-parametric trim-and-fill method to evaluate the effects of any potential missing studies on the overall results (Zhu and Carriere, 2018). We identified 4 studies, and the corresponding result was not significantly altered (OR = 1.109, 95% CI: 1.082–1.137), suggesting that our results were robust (Figure 6).

## Discussion

Our manuscript is the first systematic review and meta-analysis to focus on CF's prevalence and associated risk factors among community residents. Based on 51 studies with 123,771 cases, the pooled prevalence of CF in the model I was 16%, 95% CI (0.13–0.19), and the pooled prevalence of CF in model II was 6%, 95% CI (0.05–0.07). The pooled analysis demonstrated that engagement in age, activities, negative emotional state, especially depression appeared to be independent risk factors of CF. It was unclear whether gender or marital status were independently associated with CF. Additionally, limited evidence suggested that education level, social participation, sleeping problems, calf circumference, body fat, albumin, and vitamin D may be associated with CF. However, there appears to be no direct correlation between marital status or body mass index with CF.

Multiple instruments exist to screen for frailty, but there is no unified consensus about its predictive value and no gold standard measure utilized in clinical settings currently (Walston et al., 2018; Lee H. et al., 2020). However, there are two commonly used frailty assessment tools. One is “physical frailty,” which views frailty as a syndrome, such as the Fried criteria, whereas the other approach views frailty as a spectrum of aging, such as FI. The FI is known to predict death better than the frailty phenotype. Nevertheless, when constructing FI, the domain composed of a physical performance-based measure does not necessarily possess predictive power superior to self-reported items (Lee H. et al., 2020).

Similarly, various assessment tools are available for cognitive impairment. Because these tools have different sensitivity and validity values influenced by education, language, culture, and variable cutoffs, some findings highlight the lack of appropriate validated cognitive assessment tools. However, a screening tool is still crucial for cognitive recognition (Rosli et al., 2016; Ranjit et al., 2020). Among the studies we included, the two studies (Hao et al., 2018; Sharma et al., 2020) with the highest prevalence of CF were evaluated by FI and Fried criteria, respectively. The MMSE was used to evaluate cognition. Notably, though, the main reason for the high prevalence of CF was that the included population involved the most elderly. In contrast, studies that reported the lowest prevalence included relatively younger individuals and were found to lack data of the elderly over age 84 (Solfrizzi et al., 2017a). In addition, the vast majority of studies divide frailty into pre-frailty and frailty, which are two different severities and thus may cause variability in prevalence in these groups.

Frailty is a clinical syndrome driven by age-related biologic changes (Lee H. et al., 2020). Cognitive impairment represented by dementia is mainly a disease of the elderly (Scheltens et al., 2016; Ranjit et al., 2020). Among several geriatric syndromes, cognitive impairment and frailty are common problems in the elderly, and these two entities have a close relationship. The deterioration of one element can affect the other and may form a vicious cycle (Arai et al., 2018). The positive rate of Alzheimer's Disease-like CSF (Cerebrospinal Fluid) and amyloid lesion on PET-CT (Positron Emission Tomography-Computed Tomography) increase with age, which may be the basis for age-related pathological mechanisms of CF (Parnetti et al., 2019). Our results also demonstrated that age is an independent risk factor for CF, consistent with the above standpoints.

Our study found that people who engaged in less physical activity or social participation had a higher prevalence of CF. First, sarcopenia characterized by unintentional loss of muscle mass is a critical pathophysiological component of frailty (Shen et al., 2019). Previous studies have demonstrated that a certain degree and intensity of resistance training significantly enhanced muscle strength, muscle power, muscle morphology, and functional outcomes (Dedeyne et al., 2017; Lopez et al., 2018). A meta-analysis provided evidence that physical exercise positively affects most frail older adults (De Labra et al., 2015). Second, Scheltens conducted a study that proposed that exercise also can improve cognitive reserves (Ballard et al., 2011).

Additionally, resistance training may mitigate cognitive impairment due to evidence suggesting a positive effect in verbal fluency, cognitive flexibility, and response inhibition aspects of executive function (Zhang et al., 2020). Previous studies have demonstrated that some cognitive brain networks are disrupted in aging and cognitive disorder patients, and physical exercise may remediate the function of these brain networks effectively (Huang et al., 2016). Last but not least, people with more social participation have a higher physical activity or cognitive training opportunities. One study confirmed similar positive effects of cognitive and physical activity treatments in mitigating the cognitive decline in patients diagnosed with cognitive impairment (Fonte et al., 2019).

Our study also suggests that depression is closely related to cognitive impairment and physical frailty, consistent with recent studies (Soysal et al., 2017). First, these reciprocal associations may be shared among similar risk factors, such as cerebrovascular disease, oxidative stress, chronic inflammation, and mitochondrial dysfunction (Arai et al., 2018; Silva et al., 2019). Additionally, inflammatory cytokines may play an important role, such as interleukin-6 (IL-6), which was also elevated in individuals with CF or those with moderate to severe depression (Franceschi et al., 2000; Soysal et al., 2017). These inflammatory markers are associated with muscle strength and mass and negatively affect central dopaminergic function, resulting in fatigue, motoric slowing, depressive affect, and cognitive impairment (De Labra et al., 2015; Soysal et al., 2017). Third, mitochondrial dysfunction can be identified in numerous neurodegenerative diseases and depression, which may be an essential pathway in the pathophysiology of depression and CF (Mantzavinos and Alexiou, 2017). An influential study mentioned that depression might be a potentially treatable disorder that contributes significantly to cognitive impairment (Ballard et al., 2011). Marcos mentioned that middle-aged patients with depression displayed hippocampal atrophy and Aβ peptide deposition observed by PET-CT, indicating that protein metabolism may be altered in patients with depression (Silva et al., 2019). The importance of depression cannot be ignored when focusing on cognition and frailty in managing elderly individuals in the community.

When analyzing preventative measures, one element that can increase cognitive reserve involves education level (Silva et al., 2019). A study conducted by Philip et al. indicated that education could improve cognitive reserve (Ballard et al., 2011). Furthermore, Martin et al. analyzed the relationship between education level and cognition carefully, suggesting that the number of years of formal education completed by individuals was positively correlated with their cognitive function in adulthood and predicted a lower risk of dementia later in life (Lovden et al., 2020). Consensus guidelines for the intervention of frailty state that cognitive training is a fundamental part of frailty management (Marcucci et al., 2019). Therefore, providing more educational opportunities for the elderly in the community may be an effective measure for CF prevention.

Regarding gender, our review and analysis did not elucidate any significant difference in the prevalence of CF within community-dwelling residents. This finding is in congruence with recent systematic reviews. Shen found no significant gender differences regarding the prevalence of sarcopenia in nursing home residents (Shen et al., 2019). In another meta-analysis, Lucilla also mentioned that gender was not significantly associated with preclinical Alzheimer's Disease prevalence (Parnetti et al., 2019). This study also suggested that both elderly males and females in the community are at similar risk of developing CF.

The relationship between sleep deficits and cognitive function has been studied in detail. Omonigho proved that individuals with insomnia had a 1.65 times higher risk of developing cognitive impairment when compared to individuals without sleep problems. This study additionally estimated that approximately 15% of cognitive impairment might be attributed to sleeping problems, including insomnia (Sun et al., 2020). Hiroki reinforced this concept by reporting that patients with frailty often experience more inferior sleep quality (Nishikawa et al., 2020). A meta-analysis reported that interventions on circadian rhythms might have significant clinical implications in the frail elderly (Gallione et al., 2019). We implemented strategies to address sleep deficits that should be included in any CF preventative strategy.

Finally, nutritional status and its relation to the risk of developing CF is a salient point of discussion. Malnutrition and CF share some clinical features, such as fatigue and weight loss; therefore, it is clear that there is a correlation between them. As mentioned above, there is a distinct correlation between CF and aging. Aging is a physiological process known to produce changes in body composition, affecting the musculature and decreasing muscle volume and strength (Planella-Farrugia et al., 2019). In addition, resistance training and dietary guidance, especially foods with anti-inflammatory and antioxidant properties, can inhibit aging by reducing waist circumference and body fat percentage and increasing arm circumference and calf circumference (Lopes et al., 2020). Specific markers may be utilized to assess for malnutrition as such. Shen suggested that albumin and prealbumin rather than the body mass index may be beneficial for assessing malnutrition (Shen et al., 2019). Different studies have shown that cognitive impairment and frailty are affected by vitamin D levels (Zhou et al., 2016; Lee D. H. et al., 2020). Given the literature, there are reasons to suggest that thinner calf circumference, higher total body fat, and lower albumin content appear to be linked to a higher prevalence of CF. Body mass index alone may not be appropriate for estimating the occurrence of CF as well.

Besides, previous fall history is also a risk factor of CF. For instance, a Japanese cross-sectional survey in a total of 7,614 older people age > 70 years reported that falls associated with CF were (OR 1.132, 95% CI 1.002–1.280) (Kim et al., 2019). Another Chinese multiple-center study found that fall is an independent risk factor of CF (OR 6.653, 95% CI 2.651–16.697) (Ma et al., 2017). Furthermore, the adverse complication of fall indeed promoted sarcopenia because of the prolonged bed rest. On the other hand, fall also caused hospitalacquired pneumonia (HAP) because patients extended hospitalized days. As usual, the pathogenic microbes of HAP are multi-resistant pathogens and deteriorate the poor prognosis of CF.

## Limitation

This review has several limitations. Firstly, we only included studies written in English, which may introduce selection bias or reporting bias to our results. Secondly, our focus was on the prevalence of CF. Our analysis of all the studies involved various assessment tools related to cognition and frailty with inconsistent cutoff values. As such, this level of heterogeneity likely affected our results. However, many of these studies did provide supporting evidence regarding the validity of such assessment tools for global use. Thirdly, due to the significant heterogeneity in study designs and lack of uniformly reported risk factors in every study (such as gender, marital status, sleeping deficits), we could not perform a conglomerate meta-analysis of all risk factors of CF. However, our sensitivity analysis demonstrated that the individual study did not significantly influence the pooled results. Finally, most of the included studies were retrospective observational studies, which cannot provide a higher strength of evidence when compared to prospective cohort studies. Therefore, there are significant opportunities to expand our understanding of CF by performing further well-designed prospective cohort studies to search for effective and predictive diagnostic tools and to verify the risk factors of CF in the future.

## Conclusion/Future Direction

CF is highly prevalent in community residents. Furthermore, different definitions of CF have different prevalence rates. A multi-modal intervention for CF, including the combination of increased exercise, nutritional support, depression prevention, sleeping disorder adjustments, increased social opportunities, and multi-component strategies, may be effective for the prevention of CF. Prospective studies of a large sample size should be conducted to establish a consensus to assess CF's various reported diagnostic criteria. Additionally, the measurement of various clinical outcomes (e.g., progression to dementia, morbidity, mortality) cannot be thoroughly conducted without such studies. More well-designed randomized controlled trials are also needed to determine the types of nutritional support, the choice of exercise methods, and the measures of emotional regulation required for community residents to prevent CF.

## Data Availability Statement

The original contributions presented in the study are included in the article/Supplementary Material, further inquiries can be directed to the corresponding author/s.

## Author Contributions

YiY conceived and collected the preliminary data. TZ and YiY performed and designed the study. TZ and YR gathered and analyzed the original studies, extracted the data independently and ensured congruence with the inclusion and exclusion criteria. TZ, YR, and YiY wrote the first draft of the manuscript. TZ conceived all the figures and tables. YR and PS also independently evaluated the risk bias of all included studies. YaY completed the Supplementary Material. YW and ZL participated and guided in the discussion of an overall framework of the article. SJ and YiY corrected and validated the manuscript in its entirety. The manuscript has been read and approved by all authors.

## Conflict of Interest

The authors declare that the research was conducted in the absence of any commercial or financial relationships that could be construed as a potential conflict of interest.

## Publisher's Note

All claims expressed in this article are solely those of the authors and do not necessarily represent those of their affiliated organizations, or those of the publisher, the editors and the reviewers. Any product that may be evaluated in this article, or claim that may be made by its manufacturer, is not guaranteed or endorsed by the publisher.

## Abbreviations

CF I: Cognitive Frailty in model I
CF II: Cognitive Frailty in model II
MMSE: Mini-Mental State Examination
MoCA: Montreal Cognitive Assessment
HDS-R: Revised Hasegawa's dementia scale
CI testing: Cognitive Impairment testing
TICS-10: Telephone Interview of Cognitive Status-10 items
SPMSQ: Short Portable Mental Status Questionnaire
MiniCog: Minimal Cognition
CDR: Clinical Dementia Rating Scale.
