# Supplementary material for: Prevalence and Associated Risk Factors of Cognitive Frailty: A Systematic Review and Meta-Analysis
Source: Front Aging Neurosci. 2022 Jan 28;13:755926. doi: 10.3389/fnagi.2021.755926 (PMC8832102; doi:10.3389/fnagi.2021.755926)
Supplement: Supplementary file 1 [file Table_1.DOCX]

Supplementary Material

Supplementary Table 1: Database search

| **Pubmed** | | | **Cochrane** | | | **EMBASE** | | | **Ovid** | | | **Web of Science** | | |
| --- | --- | --- | --- | --- | --- | --- | --- | --- | --- | --- | --- | --- | --- | --- |
| **Search** | **Query** | **Items** | **Search** | **Query** | **Items** | **Search** | **Query** | **Items** | **Search** | **Query** | **Items** | **Search** | **Query** | **Items** |
| **#1** | **Frailty [MeSH Terms]** | **3461** | **#1** | **frail* ti,ab,kw** | **3635** | **#1** | **"frail*"ab,kw,ti** | **37989** | **#1** | **"frail*".ti,ab** | **8634** | **#1** | **TI=frail* ORAB=frail*** | **27093** |
| **#2** | **frail*[Title/Abstract]** | **24791** | **#2** | **cogniti* ti,ab,kw** | **76104** | **#2** | **"cogniti*"ab,kw,ti** | **591735** | **#2** | **"cogniti*"ti,ab.** | **143758** | **#2** | **TI=cogniti*ORAB=cogniti*** | **621617** |
| **#3** | **#1 OR #2** | **24968** | **#3** | **#1 AND #2** | **731** | **#3** | **#1 AND #2** | **5580** | **#3** | **#1 AND #2** | **861** | **#3** | **#1 AND #2** | **3168** |
| **#4** | **cogniti*[Title/Abstract]** | **420317** |  |  |  |  |  |  |  |  |  |  |  |  |
| **#5** | **#3 AND #4** | **3477** |  |  |  |  |  |  |  |  |  |  |  |  |

***Supplementary Table 2: Main characteristic of included studies.***

| Author/year | Country | Study Type | Age  ±SD | participants | Female | The method of frailty assessment | The method of cognitive assessment | Total | Events  CF I | Prevalence  CF Ⅰ | Events  CF Ⅱ | Prevalence  CF Ⅱ |
| --- | --- | --- | --- | --- | --- | --- | --- | --- | --- | --- | --- | --- |
| Shimada, 2018 | Japan | cross-sectional | 71.9 ±5.5 | Community | 2326 | Sarcopenia | NCGG-FAT | 4570 | 441 | 0.096 | NG | NG |
| Tsutsumimoto, 2020 | Japan | longitudinal | 73.5±5.4 | Community | 5137 | Sarcopenia | NCGG-FAT | 9936 | 1109 | 0.112 | NG | NG |
| Ma, 2017 | China | cross-sectional | ≥60 | Community | NG | FI | MMSE | 5708 | 187 | 0.033 | NG | NG |
| John, 2017 | Canada | longitudinal | 77.5 ±7.1 | Community | 1025 | FI | MMSE | 1751 | 211 | 0.121 | NG | NG |
| Hao, 2018 | China | cross-sectional | 93.6 ± 3.3 | Community | 475 | FI | MMSE | 705 | 353 | 0.501 | NG | NG |
| Li, 2020 | Taiwan | cross-sectional | ≥65 | Community | 1410 | FRAIL scale | MMSE | 2693 | 330 | 0.123 | NG | NG |
| Ruan, 2019 | China | cross-sectional | ≥60 | Community | 2766 | FRAIL scale | 3-item RCS /SCD questionnaire | 5177 | 1328 | 0.257 | NG | NG |
| Kwan, 2019 | China | cross-sectional | ≥65 | Community | 132 | FRAIL scale | CDR | 185 | 66 | 0.357 | NG | NG |
| Moon, 2019 | Korea | cross-sectional | ≥70 | Community | 1269 | FRAIL scale | TMTA，FAB，DSB，CWL | 2392 | 77 | 0.032 | NG | NG |
| Esteban-cornejo, 2019 | Spain | cohort | ≥60 | Community | 2058 | FRAIL scale | MMSE | 3677 | 832 | 0.226 | NG | NG |
| Yu, 2018 | China | longitudinal | 72.03 ± 4.9 | Community | 1691 | Fried criteria | MMSE | 3491 | 302 | 0.087 | NG | NG |
| Navarro-pardo, 2020 | Spain | cross-sectional | ≥60 | Community | 153 | Fried criteria | MoCA | 285 | 62 | 0.218 | 9 | 0.032 |
| Gifford, 2019 | US | cross-sectional | 73±7 | Community | 129 | Fried criteria | MoCA | 306 | 51 | 0.167 | NG | NG |
| Rivan, 2020 | Malaysia | longitudinal | 68.86±6.12 | Community | 443 | Fried criteria | MoCA, Digit span, RAVLT, Digit symbol, VR I, VR II | 815 | 325 | 0.399 | NG | NG |
| Jing，2020 | China | cross-sectional | 70.1±6.3 | Community | 2060 | Fried criteria | MMSE | 3242 | NG | NG | 214 | 0.066 |
| Kim, 2019 | Japan | cross-sectional | >70 | Community | 713 | Fried criteria | MMSE | 1192 | NG | NG | 25 | 0.021 |
| Roppolo, 2016 | Italian | cross-sectional | 73.6± 5.8 | Community | 380 | Fried criteria | MMSE | 594 | NG | NG | 26 | 0.044 |
| Sharma, 2020 | Indian | cross-sectional | 83.34±3.93 | Community | 124 | Fried criteria | MMSE | 200 | NG | NG | 116 | 0.580 |
| Xie, 2020 | China | cross-sectional | 81.38 ± 4.7 | Community | 922 | Fried criteria | MMSE | 1585 | NG | NG | 114 | 0.072 |
| Ge, 2019 | U.S. | longitudinal | ≥65 | Community | 4361 | Fried criteria | Executive function or memory, diagnosis of dementia or AD/AD8 Dementia Screening Interview | 7497 | NG | NG | 903 | 0.120 |
| Li, 2019 | China | cohort | 75.33 ± 3.9 | Community | 936 | Fried criteria | HDS-R | 1757 | NG | NG | 341 | 0.194 |
| Ma, 2021 | China | longitudinal | 75.2±3.7 | Community | 840 | Fried criteria | HDS-R | 1606 | NG | NG | 25 | 0.016 |
| Wanaratna, 2019 | Thailand | cross-sectional | 69.4±6.9 | Community | 600 | Fried criteria | MiniCog | 780 | NG | NG | 19 | 0.024 |
| Ju¨rschik, 2012 | Spain | cross-sectional | ≥75 | Community | NG | Fried criteria | Pfeiffer’s Test | 523 | NG | NG | 10 | 0.019 |
| Nyunt, 2017 | Singapore | longitudinal | ≥55 | Community | 1213 | Fried criteria | MoCA | 1938 | 87 | 0.045 | 22 | 0.011 |
| Liu, 2018 | US | longitudinal | ≥70 | Community | 451 | Fried criteria | MMSE | 690 | NG | NG | 45 | 0.065 |
| Ahmad, 2018 | Malaysia | longitudinal | ≥60 | Community | 1437 | Fried criteria | MMSE | 2324 | 92 | 0.040 | 43 | 0.018 |
| Alencar, 2012 | Brazil | cohort | ≥65 | Community | 159 | Fried criteria | MMSE | 207 | 56 | 0.271 | 28 | 0.135 |
| Feng, 2017 | Singapore | longitudinal | 65.8 ±7.46 | Community | 1037 | Fried criteria | MMSE | 1575 | 64 | 0.041 | 12 | 0.008 |
| Macuco, 2012 | Brazil | cross-sectional | ≥65 | Community | 258 | Fried criteria | MMSE | 384 | 59 | 0.154 | 12 | 0.031 |
| A´vila-Funes, 2009 | French | longitudinal | 74.1 ± 5.2 | Community | 3690 | Fried criteria | MMSE | 6030 | 437 | 0.072 | 92 | 0.015 |
| Delrieu, 2016 | France | RCT | 75.37±4.4 | Community | 1046 | Fried criteria | CDR | 1617 | NG | NG | 356 | 0.220 |
| Downer, 2019 | US | longitudinal | 82.2 ±3.7 | Community | 378 | Fried criteria | MMSE | 639 | 81 | 0.127 | NG | NG |
| Bunce, 2019 | Australia | longitudinal | ≥70 | Community | 440 | Fried criteria | MMSE | 878 | 64 | 0.073 | 22 | 0.025 |
| Montero-odasso, 2016 | UK | cohort | 76.7 ±8.6 | Community | 158 | Fried criteria | MoCA | 252 | 94 | 0.373 | 27 | 0.107 |
| Hoogendijk, 2014 | Netherland | longitudinal | ≥65 | Community | NG | Fried criteria | MMSE | 1205 | NG | NG | 27 | 0.022 |
| Solfrizzi, 2017 | Italian | longitudinal | 65-84 | Community | 1333 | Fried criteria | MMSE | 2373 | NG | NG | 17 | 0.007 |
| Chu, 2019 | US | cohort | 73.2 ±6.8 | Community | 2178 | Fried criteria | CI test | 3848 | NG | NG | 308 | 0.080 |
| Aliberti, 2019 | US | cohort | 74.4 ±7.0 | Community | 4098 | Fried criteria | HRS self-respondents | 7338 | NG | NG | 397 | 0.054 |
| Thein, 2018 | China | longitudinal | ≥55 | Community | 1693 | Fried criteria | MMSE | 2696 | NG | NG | 64 | 0.024 |
| Bello-chavolla, 2017 | Mexican | cross-sectional | 77.7±5.8 | Community | 73 | Fried criteria | MMSE | 135 | NG | NG | 5 | 0.037 |
| Chen, 2020 | China | longitudinal | 68.1 ± 6.5 | Community | 2479 | Fried criteria | TICS-10 | 5113 | NG | NG | 82 | 0.016 |
| Brigola, 2019 | Brazil | cross-sectional | 71.3 ±7.8 | Community | 366 | Fried criteria | MMSE | 667 | 200 | 0.300 | 88 | 0.132 |
| Ge, 2020 | China | cross-sectional | 67.8 ± 5.9 | Community | 2408 | Fried criteria | SPMSQ | 4103 | 117 | 0.029 | 117 | 0.029 |
| Liu, 2017 | Taiwan | longitudinal | 73.3±5.3 | Community | 314 | Fried criteria | MMSE | 678 | 90 | 0.133 | 90 | 0.133 |
| Llano, 2019 | Brazil | cross-sectional | ≥60 | Community | 460 | Self-reported component of frailty | MMSE | 820 | 318 | 0.388 | NG | NG |
| Wang, 2019 | China | cross-sectional | ≥60 | Community | 1831 | SPPB | MMSE | 3157 | 89 | 0.028 | NG | NG |
| Silva, 2020 | Brazil | cross-sectional | ≥60 | Community | 93 | IVCF-20 | MMSE | 305 | 35 | 0.115 | NG | NG |
| Arnadottir, 2020 | England | cohort | 78±5.7 | Community | 5110 | The Strawbridge questionnaire | cognitive domains | 9803 | 1684 | 0.172 | NG | NG |
| Lee, 2018 | Taiwan | longitudinal | 65.1±9.5 | Community | 510 | Sarcopenia | SPMSQ | 1103 | 95 | 0.086 | NG | NG |
| Katayama, 2020 | Japan | cross-sectional | 72.5 ±6.1 | Community | 4489 | Sarcopenia | NCGG-FAT | 8003 | 853 | 0.107 | NG | NG |

***Abbreviation***

SBPP: Short Performance Physical Battery (SPPB) score

IVCF-20: Clinical-Functional Vulnerability Index-20

AD: Alzheimer’s disease

TMT: Trail Making Test Part A

FAB: Frontal Assessment Battery

DSB: Digit Span Backward test

CWL: Word List Recall test

RCS: Rapid Cognitive Screen tools

CF I: Cognitive Frailty in model I

CF II: Cognitive Frailty in model II

MMSE: Mini-Mental State Examination

MoCA: Montreal Cognitive Assessment

HDS-R: Revised Hasegawa’s dementia scale

CI testing: Cognitive Impairment testing

TICS-10: Telephone Interview of Cognitive Status-10 items

SPMSQ: Short Portable Mental Status Questionnaire

MiniCog: Minimal Cognition

CDR: Clinical Dementia Rating Scale

NCGG-FAT: National Center for Geriatrics and Gerontology functional assessment tool

HRS: Health and Retirement Study

RAVLT: Rey Auditory Verbal Learning Test

VR I: Visual Reproduction I

VR II: Visual Reproduction II

FI: Frailty Index

***Supplementary Table 3: Associated risk factor of CF in the included study.***

***Associated factor: Education***

| Author/year | Effect measure | Adjusted OR/RR/HR  (95%CI) | Lower Confidence Interval | Upper Confidence Interval | Adjusted P value | Characteristics | Reference |
| --- | --- | --- | --- | --- | --- | --- | --- |
| Katayama，2020 | OR | 0.95 | 0.91 | 0.99 | 0.013 | - | ≤6 y |
| Navarro-Pardo,  2020 | OR | 3.43 | 1.63 | 7.20 | 0.01 | - | more than 7 years of education |
| Chu,2019 | HR | 0.705 | 0.423 | 1.175 | 0.765 | 9th–12th Grade (no diploma) | 8th Grade or Less |
| Chu,2019 | HR | 0.499 | 0.317 | 0.785 | <0.05 | High School Diploma or Equivalent | 8th Grade or Less |
| Chu,2019 | HR | 0.583 | 0.365 | 0.931 | <0.05 | Some College but no Degree | 8th Grade or Less |
| Chu,2019 | HR | 0.343 | 0.199 | 0.590 | <0.05 | Associates or Bachelor’s Degree | 8th Grade or Less |
| Chu,2019 | HR | 0.353 | 0.188 | 0.665 | <0.05 | Graduate Degree | 8th Grade or Less |
| RUAN,2019 | OR | 0.583 | 0.404 | 0.84 | 0.004 | 6-12 | ≤6 y |
| RUAN,2019 | OR | 1.004 | 0.647 | 1.558 | 0.986 | ≥15 | ≤6 y |

***Associated factor: Age***

| Author/year | Effect measure | Adjusted OR/RR/HR  (95%CI) | Lower Confidence Interval | Upper Confidence Interval | Adjusted P value | Characteristics | Reference |
| --- | --- | --- | --- | --- | --- | --- | --- |
| Ruan,2019 | OR | 2.928 | 2.03 | 4.222 | 0.000 | - | 70-79y，60-69y |
| Ruan,2019 | OR | 19.708 | 13.492 | 28.788 | 0.000 | - | ＞80y，60-69y |
| Navarro-Pardo,  2020 | OR | 0.99 | 0.31 | 3.15 | >0.05 | 65–69 | 60-64y |
| Navarro-Pardo,  2020 | OR | 2.10 | 0.74 | 5.96 | >0.05 | 70–74 | 60-64y |
| Navarro-Pardo,  2020 | OR | 1.21 | 0.40 | 3.67 | >0.05 | 75–79 | 60-64y |
| Navarro-Pardo,  2020 | OR | 4.24 | 1.57 | 11.44 | < 0.05 | 80+ | ＞80y，60-64y |
| Ma,2017 | OR | 4.918 | 1.845 | 13.107 | 0.001 | - | ＜75 years |
| Xie,2020 | OR | 1.99 | 1.10 | 3.59 | 0.023 | - | 81–85y,75–80y |
| Xie,2020 | OR | 6.43 | 3.66 | 11.29 | <.001 | - | 86 years or older,75–80y |
| Kim,2019 | OR | 1.151 | 1.053 | 1.257 | < 0.05 | - | Age (per 1 year) |
| Katayama,2020 | OR | 1.06 | 1.05 | 1.08 | <.001 | - | Age (per 1 year) |
| Rivan,2020 | OR | 1.123 | 1.044 | 1.208 | 0.002 | - | Age (per 1 year) |
| Chu,2019 | HR | 1.108 | 1.076 | 1.141 | < 0.05 | Age spline covariate 1 | Baseline age (knots at 75, 85, and 95 y) |
| Chu,2019 | HR | 0.950 | 0.891 | 1.013 | >0.05 | Age spline covariate 2 | Baseline age (knots at 75, 85, and 95 y) |

***Associated factor: Sex***

| Author/year | Effect measure | Adjusted OR/RR/HR (95%CI) | Lower Confidence Interval | Upper Confidence Interval | Adjusted P value | Characteristics | Reference |
| --- | --- | --- | --- | --- | --- | --- | --- |
| Katayama,2020 | OR | 0.26 | 0.18 | 0.39 | <0.001 | Female | Male |
| Chu,2019 | HR | 1.009 | 0.773 | 1.316 | >0.05 | Female | Male |

***Associated factor: Marital status***

| Author/year | Effect measure | Adjusted OR/RR/HR (95%CI) | Lower Confidence Interval | Upper Confidence Interval | Adjusted P value | Characteristics | Reference |
| --- | --- | --- | --- | --- | --- | --- | --- |
| RUAN,2019 | OR | 0.995 | 0.327 | 3.025 | 0.993 | Married | Single |
| RUAN,2019 | OR | 1.802 | 0.564 | 5.757 | 0.32 | Widowed | Single |

***Associated factor: Social participation***

| Author/year | Effect measure | Adjusted OR/RR/HR (95%CI) | Lower Confidence Interval | Upper Confidence Interval | Adjusted P value | Characteristics | Reference |
| --- | --- | --- | --- | --- | --- | --- | --- |
| Xie,2020 | OR | 0.61 | 0.39 | 0.96 | 0.034 | More social activities | Less social  activities |

***Associated factor: Depression***

| Author/year | Effect measure | Adjusted OR/RR/HR  (95%CI) | Lower Confidence Interval | Upper Confidence Interval | Adjusted P value | Assessment tools |
| --- | --- | --- | --- | --- | --- | --- |
| Li-Kuo Liu, 2017 | OR | 1.077 | 1.03 | 1.125 | 0.001 | CES-D score |
| Katayama,2020 | OR | 1.12 | 1.09 | 1.16 | <.001 | GDS score |
| Rivan,2020 | OR | 1.200 | 1.051 | 1.369 | 0.007 | GDS score |
| Xie,2020 | OR | 3.88 | 2.39 | 6.29 | <.001 | GDS score |
| Chu,2019 | HR | 1.350 | 0.805 | 2.266 | >0.05 | PHQT |
| Li,2020 | RR | 5.63 | 3.83 | 8.28 | < 0.001 | EQ-5D |
| Navarro-Pardo，2020 | OR | 2.94 | 1.35 | 6.39 | 0.001 | GHQ12 |

***Associated factor: Sleep problem***

| Author/year | Effect measure | Adjusted OR/RR/HR (95%CI) | Lower Confidence Interval | Upper Confidence Interval | Adjusted P value | Characteristics | Reference |
| --- | --- | --- | --- | --- | --- | --- | --- |
| Xie,2020 | OR | 1.84 | 1.07 | 3.17 | 0.028 | insomnia occasionally | Good sleep |
| Xie,2020 | OR | 2.38 | 1.33 | 4.26 | 0.004 | insomnia everyday | Good sleep |

***Associated factor: Activity***

| Author/year | Effect measure | Adjusted OR/RR/HR (95%CI) | Lower Confidence Interval | Upper Confidence Interval | Adjusted P value | Characteristics | Reference |
| --- | --- | --- | --- | --- | --- | --- | --- |

| Katayama,2020 | OR | 1.76 | 1.47 | 2.11 | <.001 | Less going-out activity | More activities |
| --- | --- | --- | --- | --- | --- | --- | --- |
| Katayama,2020 | OR | 2.80 | 1.97 | 3.97 | <.001 | Less cognitive activities | More activities |
| Katayama,2020 | OR | 3.94 | 2.58 | 6.03 | <.001 | Less multidomain activities | More activities |
| Xie,2020 | OR | 2.69 | 1.66 | 4.34 | <.001 | Less activities | Sedentary lifestyle |

***Associated factor: Nutrition***

| Author/year | Effect measure | Adjusted OR/RR/HR (95%CI) | Lower Confidence Interval | Upper Confidence Interval | Adjusted P value | Assessment tools or evaluating indicators |
| --- | --- | --- | --- | --- | --- | --- |
| Li-Kuo Liu,2017 | OR | 0.869 | 0.766 | 0.986 | 0.030 | MNA |
| Kim,2019 | OR | 0.736 | 0.628 | 0.863 | <0.001 | CNAQ (council on nutrition appetite questionnaire) (per 1 unit) |
| Kim,2019 | OR | 0.748 | 0.625 | 0.895 | <0.05 | Calf circumference (per 1 unit) |
| Katayama,2020 | OR | 1.04 | 1.01 | 1.07 | 0.003 | Total body fat |
| Katayama,2020 | OR | 0.82 | 0.78 | 0.87 | <.001 | ASM, kg |
| Katayama,2020 | OR | 0.45 | 0.34 | 0.59 | 0<.001 | Albumin, mg/dL |
| Katayama,2020 | OR | 1.05 | 0.98 | 1.11 | 0.162 | BMI |
| Rivan,2020 | OR | 0.362 | 0.141 | 0.930 | 0.035 | Vitamin D (µg, continuous) |

***Supplementary Table 4: The (bias)quality assessment of included studies.***

| **first author/year** | **1.Was the study’s target population a close representation of the national population in relation to relevant variables?** | **2. Was the sampling frame a true or close representation of the target population?** | **3. Was some form of random selection used to select the sample, OR was a census undertaken?** | **4.Was the likelihood of nonresponse bias minimal?** | **5. Were data collected directly from the subjects (as opposed to a proxy)?** | **6.Was an acceptable case definition used in the study?** | **7. Was the study instrument that measured the parameter of interest shown to have validity and reliability?** | **8. Was the same mode of data collection used for all subjects?** | **9. Was the length of the shortest prevalence period for the parameter of interest appropriate** | **10. Were the numerator(s) and de0minator(s) for the parameter of interest appropriate?** | **All item scores** |
| --- | --- | --- | --- | --- | --- | --- | --- | --- | --- | --- | --- |
| Shimada,2018 | 1 | 1 | 1 | 1 | 1 | 1 | 1 | 1 | 1 | 1 | 10 |
| Tsutsumimoto, 2020 | 1 | 1 | 1 | 1 | 1 | 1 | 1 | 1 | 1 | 1 | 10 |
| Ma, 2017 | 0 | 0 | 0 | 1 | 1 | 1 | 1 | 1 | 1 | 1 | 7 |
| John, 2017 | 0 | 1 | 1 | 1 | 1 | 1 |  | 1 | 1 | 1 | 8 |
| Hao, 2018 | 0 | 0 | 0 | 1 | 1 | 1 | 1 | 1 | 0 | 1 | 6 |
| Li, 2020 | 0 | 0 | 0 | 0 | 1 | 1 | 1 | 1 | 1 | 1 | 6 |
| Ruan, 2019 | 0 | 1 | 1 | 1 | 1 | 1 | 1 | 1 | 1 | 1 | 9 |
| Kwan, 2019 | 0 | 0 | 0 | 1 | 1 | 1 | 1 | 1 | 1 | 1 | 7 |
| Moon, 2019 | 1 | 1 | 1 | 1 | 1 | 1 | 1 | 1 | 1 | 1 | 10 |
| Esteban-cornejo, 2019 | 1 | 1 | 1 | 0 | 1 | 1 | 1 | 1 | 1 | 1 | 9 |
| Yu, 2018 | 1 | 1 | 1 | 1 | 1 | 1 | 1 | 1 | 1 | 1 | 10 |
| Navarro-pardo, 2020 | 1 | 1 | 0 | 1 | 1 | 1 | 1 | 1 | 1 | 1 | 9 |
| Gifford, 2019 | 0 | 1 | 0 | 0 | 1 | 1 | 1 | 1 | 1 | 1 | 7 |
| Rivan, 2020 | 0 | 0 | 1 | 1 | 1 | 1 | 1 | 1 | 1 | 1 | 8 |
| Jing，2020 | 0 | 0 | 1 | 0 | 1 | 1 | 1 | 1 | 1 | 1 | 7 |
| Kim, 2019 | 0 | 1 | 0 | 0 | 1 | 1 | 1 | 1 | 1 | 1 | 7 |
| Roppolo, 2016 | 0 | 0 | 0 | 0 |  | 1 | 1 | 1 | 1 | 1 | 5 |
| Sharma, 2020 | 0 | 0 | 1 | 1 | 1 | 1 | 1 | 1 | 1 | 1 | 8 |
| Xie, 2020 | 0 | 1 | 1 | 0 | 1 | 1 | 1 | 1 | 1 | 1 | 8 |
| Ge, 2019 | 1 | 1 | 0 | 0 | 1 | 1 | 1 | 1 | 1 | 1 | 8 |
| Li, 2019 | 0 | 0 | 0 | 0 | 1 | 1 | 1 | 1 | 1 | 1 | 6 |
| Ma, 2021 | 1 | 1 | 1 | 0 | 1 | 1 | 1 | 1 | 1 | 1 | 9 |
| Wanaratna, 2019 | 0 | 1 | 0 | 1 | 1 | 1 | 1 | 1 | 1 | 1 | 8 |
| Ju¨rschik, 2012 | 0 | 1 | 1 | 0 | 1 | 1 | 1 | 1 | 1 | 1 | 8 |
| Nyunt, 2017 | 1 | 1 | 1 | 0 | 1 | 1 | 1 | 0 | 1 | 1 | 8 |
| Liu, 2018 | 0 | 1 | 1 | 0 | 1 | 1 | 1 | 1 | 0 | 1 | 7 |
| Ahmad, 2018 | 0 | 0 | 1 | 1 | 1 | 1 | 1 | 1 | 1 | 1 | 8 |
| Alencar, 2012 | 0 | 0 | 1 | 1 | 1 | 1 | 1 | 1 | 1 | 1 | 8 |
| Feng, 2017 | 1 | 1 | 1 | 0 | 1 | 1 | 1 | 1 | 1 | 1 | 9 |
| Macuco, 2012 | 0 | 0 | 0 | 1 | 1 | 1 | 1 | 1 | 1 | 1 | 7 |
| A´vila-Funes, 2009 | 1 | 1 | 1 | 0 | 1 | 1 | 1 | 1 | 1 | 1 | 9 |
| Delrieu, 2016 | 1 | 1 | 1 | 1 | 1 | 1 | 1 | 1 | 1 | 1 | 10 |
| Downer, 2019 | 0 | 0 | 1 | 0 | 0 | 1 | 1 | 1 | 1 | 1 | 6 |
| Bunce, 2019 | 0 | 0 | 0 | 0 | 1 | 1 | 1 | 1 | 1 | 1 | 6 |
| Montero-odasso, 2016 | 0 | 1 | 0 | 1 | 1 | 1 | 1 | 1 | 0 | 1 | 7 |
| Hoogendijk, 2014 | 1 | 1 | 0 | 1 | 1 | 1 | 1 | 1 | 0 | 1 | 8 |
| Solfrizzi, 2017 | 1 | 1 | 1 | 0 | 1 | 1 | 1 | 1 | 1 | 1 | 9 |
| Chu, 2019 | 1 | 1 | 1 | 0 | 1 | 1 | 1 | 1 | 1 | 1 | 9 |
| Aliberti, 2019 | 1 | 1 | 1 | 1 | 1 | 1 | 1 | 1 | 1 | 1 | 10 |
| Thein, 2018 | 1 | 1 | 1 | 0 | 1 | 1 | 1 | 1 | 1 | 1 | 9 |
| Bello-chavolla, 2017 | 0 | 1 | 1 | 1 | 1 | 1 | 1 | 1 | 1 | 1 | 9 |
| Chen, 2020 | 0 | 1 | 1 | 1 | 1 | 1 | 1 | 1 | 1 | 1 | 9 |
| Brigola, 2019 | 0 | 0 | 0 | 0 | 1 | 1 | 1 | 1 | 1 | 1 | 6 |
| Ge, 2020 | 1 | 1 | 1 | 1 | 1 | 1 | 1 | 1 | 1 | 1 | 10 |
| Liu, 2017 | 0 | 1 | 0 | 1 | 1 | 1 | 1 | 1 | 0 | 1 | 7 |
| Llano, 2019 | 0 | 0 | 1 | 0 | 1 | 1 | 1 | 1 | 1 | 1 | 7 |
| Wang, 2019 | 0 | 0 | 1 | 1 | 1 | 1 | 1 | 1 | 0 | 1 | 7 |
| Silva, 2020 | 1 | 1 | 1 | 1 | 1 | 1 | 1 | 1 | 1 | 1 | 10 |
| Arnadottir, 2020 | 1 | 1 | 1 | 1 | 1 | 1 | 1 | 1 | 1 | 1 | 10 |
| Lee, 2018 | 0 | 1 | 0 | 0 | 1 | 1 | 1 | 1 | 1 | 1 | 7 |
| Katayama, 2020 | 0 | 1 | 0 | 0 | 1 | 1 | 1 | 1 | 1 | 1 | 7 |

***Supplementary Material 5: All included studies in this systematic review and meta-analysis.***

1. Ahmad, N. S., Hairi, N. N., Said, M. A., Kamaruzzaman, S. B., Choo, W. Y., Hairi, F., et al. (2018). Prevalence, transitions and factors predicting transition between frailty states among rural community-dwelling older adults in Malaysia. PLoS One. 13, e0206445. doi: 10.1371/journal.pone.0206445

2. Alencar, M. A., Dias, J. M., Figueiredo, L. C. and Dias, R. C. (2013). Frailty and cognitive impairment among community-dwelling elderly. Arq. Neuropsiquiatr. 71, 362-7. doi: 10.1590/0004-282X20130039

3. Aliberti, M. J. R., Cenzer, I. S., Smith, A. K., Lee, S. J., Yaffe, K. and Covinsky, K. E. (2019). Assessing Risk for Adverse Outcomes in Older Adults: The Need to Include Both Physical Frailty and Cognition. J. Am. Geriatr. Soc. 67, 477-483. doi: 10.1111/jgs.15683

4. Arnadottir, S. A., Bruce, J., Lall, R., Withers, E. J., Underwood, M., Shaw, F., et al. (2020). The importance of different frailty domains in a population based sample in England. BMC Geriatr. 20, 16. doi: 10.1186/s12877-019-1411-9

5. Avila-Funes, J. A., Amieva, H., Barberger-Gateau, P., Le Goff, M., Raoux, N., Ritchie, K., et al. (2009). Cognitive impairment improves the predictive validity of the phenotype of frailty for adverse health outcomes: the three-city study. J. Am. Geriatr. Soc. 57, 453-61. doi: 10.1111/j.1532-5415.2008.02136.x

6.Bello-Chavolla, O. Y., Aguilar-Salinas, C. A. and Avila-Funes, J. A. (2017). Geriatric Syndromes and Not Cardiovascular Risk Factors are Associated with Cognitive Impairment among Mexican Community-Dwelling Elderly with Type 2 Diabetes. Rev Invest Clin. 69, 166-172. doi: 10.24875/ric.17002169

7. Brigola, A. G., Ottaviani, A. C., Carvalho, D. H. T., Oliveira, N. A., Souza, E. N. and Pavarini, S. C. I. (2020). Association between cognitive impairment and criteria for frailty syndrome among older adults. Arq. Neuropsiquiatr. 78, 2-8. doi: 10.1590/0004-282X20190138

8.Bunce, D., Batterham, P. J. and Mackinnon, A. J. (2019). Long-term Associations Between Physical Frailty and Performance in Specific Cognitive Domains. J Gerontol B Psychol Sci Soc Sci. 74, 919-926. doi: 10.1093/geronb/gbx177

9. Chen, C., Park, J., Wu, C., Xue, Q., Agogo, G., Han, L., et al. (2020). Cognitive frailty in relation to adverse health outcomes independent of multimorbidity: results from the China health and retirement longitudinal study. Aging (Albany NY). 12, 23129-23145. doi: 10.18632/aging.104078

10. Chu, N. M., Bandeen-Roche, K., Tian, J., Kasper, J. D., Gross, A. L., Carlson, M. C., et al. (2019). Hierarchical Development of Frailty and Cognitive Impairment: Clues Into Etiological Pathways. J Gerontol A Biol Sci Med Sci. 74, 1761-1770. doi: 10.1093/gerona/glz134

11. Delrieu, J., Andrieu, S., Pahor, M., Cantet, C., Cesari, M., Ousset, P. J., et al. (2016). Neuropsychological Profile of "Cognitive Frailty" Subjects in MAPT Study. J Prev Alzheimers Dis. 3, 151-159. doi: 10.14283/jpad.2016.94

12. Downer, B., Al Snih, S., Howrey, B. T., Raji, M. A., Markides, K. S. and Ottenbacher, K. J. (2019). Combined effects of cognitive impairment and pre-frailty on future frailty and death in older Mexican Americans. Aging Ment Health. 23, 1405-1412. doi: 10.1080/13607863.2018.1493719

13. Esteban-Cornejo, I., Cabanas-Sanchez, V., Higueras-Fresnillo, S., Ortega, F. B., Kramer, A. F., Rodriguez-Artalejo, F., et al. (2019). Cognitive Frailty and Mortality in a National Cohort of Older Adults: the Role of Physical Activity. Mayo Clin. Proc. 94, 1180-1189. doi: 10.1016/j.mayocp.2018.10.027

14. Feng, L., Nyunt, M. S., Gao, Q., Feng, L., Lee, T. S., Tsoi, T., et al. (2017). Physical Frailty, Cognitive Impairment, and the Risk of Neurocognitive Disorder in the Singapore Longitudinal Ageing Studies. J Gerontol A Biol Sci Med Sci. 72, 369-375. doi: 10.1093/gerona/glw050

15. Ge, M., Zhang, Y., Zhao, W., Yue, J., Hou, L., Xia, X., et al. (2020). Prevalence and Its Associated Factors of Physical Frailty and Cognitive Impairment: Findings from the West China Health and Aging Trend Study (WCHAT). J. Nutr. Health. Aging. 24, 525-533. doi: 10.1007/s12603-020-1363-y

16. Ge, M. L., Carlson, M. C., Bandeen-Roche, K., Chu, N. M., Tian, J., Kasper, J. D., et al. (2020). U.S. National Profile of Older Adults with Cognitive Impairment Alone, Physical Frailty Alone, and Both. J. Am. Geriatr. Soc. 68, 2822-2830. doi: 10.1111/jgs.16769

17. Gifford, K. A., Bell, S. P., Liu, D., Neal, J. E., Turchan, M., Shah, A. S., et al. (2019). Frailty Is Related to Subjective Cognitive Decline in Older Women without Dementia. J. Am. Geriatr. Soc. 67, 1803-1811. doi: 10.1111/jgs.15972

18. Hao, Q., Dong, B., Yang, M., Dong, B. and Wei, Y. (2018). Frailty and Cognitive Impairment in Predicting Mortality Among Oldest-Old People. Front. Aging Neurosci. 10, 295. doi: 10.3389/fnagi.2018.00295

19. Hoogendijk, E. O., Van Hout, H. P., Heymans, M. W., Van Der Horst, H. E., Frijters, D. H., Broese Van Groenou, M. I., et al. (2014). Explaining the association between educational level and frailty in older adults: results from a 13-year longitudinal study in the Netherlands. Ann. Epidemiol. 24, 538-44 e2. doi: 10.1016/j.annepidem.2014.05.002

20. Jing, Z., Li, J., Wang, Y., Ding, L., Tang, X., Feng, Y., et al. (2020). The mediating effect of psychological distress on cognitive function and physical frailty among the elderly: Evidence from rural Shandong, China. J. Affect. Disord. 268, 88-94. doi: 10.1016/j.jad.2020.03.012

21. Jurschik, P., Nunin, C., Botigue, T., Escobar, M. A., Lavedan, A. and Viladrosa, M. (2012). Prevalence of frailty and factors associated with frailty in the elderly population of Lleida, Spain: the FRALLE survey. Arch. Gerontol. Geriatr. 55, 625-31. doi: 10.1016/j.archger.2012.07.002

22. Katayama, O., Lee, S., Bae, S., Makino, K., Shinkai, Y., Chiba, I., et al. (2021). Lifestyle Activity Patterns Related to Physical Frailty and Cognitive Impairment in Urban Community-Dwelling Older Adults in Japan. J. Am. Med. Dir. Assoc. 22, 583-589. doi: 10.1016/j.jamda.2020.05.031

23. Kim, H., Awata, S., Watanabe, Y., Kojima, N., Osuka, Y., Motokawa, K., et al. (2019). Cognitive frailty in community-dwelling older Japanese people: Prevalence and its association with falls. Geriatr Gerontol Int. 19, 647-653. doi: 10.1111/ggi.13685

24. Kwan, R. Y. C., Leung, A. Y. M., Yee, A., Lau, L. T., Xu, X. Y. and Dai, D. L. K. (2019). Cognitive Frailty and Its Association with Nutrition and Depression in Community-Dwelling Older People. J. Nutr. Health. Aging. 23, 943-948. doi: 10.1007/s12603-019-1258-y

25. Lee, W. J., Peng, L. N., Liang, C. K., Loh, C. H. and Chen, L. K. (2018). Cognitive frailty predicting all-cause mortality among community-living older adults in Taiwan: A 4-year nationwide population-based cohort study. PLoS One. 13, e0200447. doi: 10.1371/journal.pone.0200447

26. Li, C. L., Chang, H. Y. and Stanaway, F. F. (2020). Combined effects of frailty status and cognitive impairment on health-related quality of life among community dwelling older adults. Arch. Gerontol. Geriatr. 87, 103999. doi: 10.1016/j.archger.2019.103999

27. Li, Y. X., Jiang, X. Y., Stone, C., Ma, Y. J., Liu, Q., Hu, Z. H., et al. (2019). A new physical-cognitive scale for assessment of frailty in Chinese Han elderly. Neurol. Res. 41, 728-733. doi: 10.1080/01616412.2019.1609164

28. Liu, L. K., Chen, C. H., Lee, W. J., Wu, Y. H., Hwang, A. C., Lin, M. H., et al. (2018). Cognitive Frailty and Its Association with All-Cause Mortality Among Community-Dwelling Older Adults in Taiwan: Results from I-Lan Longitudinal Aging Study. Rejuvenation Res. 21, 510-517. doi: 10.1089/rej.2017.2038

29. Liu, Z., Han, L., Gahbauer, E. A., Allore, H. G. and Gill, T. M. (2018). Joint Trajectories of Cognition and Frailty and Associated Burden of Patient-Reported Outcomes. J. Am. Med. Dir. Assoc. 19, 304-309 e2. doi: 10.1016/j.jamda.2017.10.010

30. Llano, P. M. P., Lange, C., Sequeira, C., Jardim, V., Castro, D. S. P. and Santos, F. (2019). Factors associated with frailty syndrome in the rural elderly. Rev Bras Enferm. 72, 14-21. doi: 10.1590/0034-7167-2017-0079

31. Ma, L., Zhang, L., Zhang, Y., Li, Y., Tang, Z. and Chan, P. (2017). Cognitive Frailty in China: Results from China Comprehensive Geriatric Assessment Study. Front Med (Lausanne). 4, 174. doi: 10.3389/fmed.2017.00174

32. Ma, Y., Li, X., Pan, Y., Zhao, R., Wang, X., Jiang, X., et al. (2021). Cognitive frailty and falls in Chinese elderly people: a population-based longitudinal study. Eur. J. Neurol. 28, 381-388. doi: 10.1111/ene.14572

33. Macuco, C. R., Batistoni, S. S., Lopes, A., Cachioni, M., Da Silva Falcao, D. V., Neri, A. L., et al. (2012). Mini-Mental State Examination performance in frail, pre-frail, and non-frail community dwelling older adults in Ermelino Matarazzo, Sao Paulo, Brazil. Int. Psychogeriatr. 24, 1725-31. doi: 10.1017/S1041610212000907

34. Montero-Odasso, M. M., Barnes, B., Speechley, M., Muir Hunter, S. W., Doherty, T. J., Duque, G., et al. (2016). Disentangling Cognitive-Frailty: Results From the Gait and Brain Study. J Gerontol A Biol Sci Med Sci. 71, 1476-1482. doi: 10.1093/gerona/glw044

35. Moon, J. H., Huh, J. S., Won, C. W. and Kim, H. J. (2019). Is Polypharmacy Associated with Cognitive Frailty in the Elderly? Results from the Korean Frailty and Aging Cohort Study. J. Nutr. Health. Aging. 23, 958-965. doi: 10.1007/s12603-019-1274-y

36. Navarro-Pardo, E., Facal, D., Campos-Magdaleno, M., Pereiro, A. X. and Juncos-Rabadan, O. (2020). Prevalence of Cognitive Frailty, Do Psychosocial-Related Factors Matter? Brain Sci. 10, doi: 10.3390/brainsci10120968

37. Nyunt, M. S. Z., Soh, C. Y., Gao, Q., Gwee, X., Ling, A. S. L., Lim, W. S., et al. (2017). Characterisation of Physical Frailty and Associated Physical and Functional Impairments in Mild Cognitive Impairment. Front Med (Lausanne). 4, 230. doi: 10.3389/fmed.2017.00230

38. Rivan, N. F. M., Shahar, S., Rajab, N. F., Singh, D. K. A., Che Din, N., Mahadzir, H., et al. (2020). Incidence and Predictors of Cognitive Frailty Among Older Adults: A Community-based Longitudinal Study. Int. J. Env. Res. Public Health. 17, doi: 10.3390/ijerph17051547

39. Roppolo, M., Mulasso, A. and Rabaglietti, E. (2017). Cognitive Frailty in Italian Community-Dwelling Older Adults: Prevalence Rate and Its Association with Disability. Int. J. Environ. Res. Public. Health. 21, 631-636. doi: 10.1007/s12603-016-0828-5

40. Ruan, Q., Xiao, F., Gong, K., Zhang, W., Zhang, M., Ruan, J., et al. (2020). Prevalence of Cognitive Frailty Phenotypes and Associated Factors in a Community-Dwelling Elderly Population. #N/A. 24, 172-180. doi: 10.1007/s12603-019-1286-7

41. Sharma, P. K., Reddy, B. M. and Ganguly, E. (2020). Frailty Syndrome among oldest old Individuals, aged >/=80 years: Prevalence & Correlates. J Frailty Sarcopenia Falls. 5, 92-101. doi: 10.22540/JFSF-05-092

42. Shimada, H., Doi, T., Lee, S., Makizako, H., Chen, L. K. and Arai, H. (2018). Cognitive Frailty Predicts Incident Dementia among Community-Dwelling Older People. J Clin Med. 7, doi: 10.3390/jcm7090250

43. Silva, J., Leite, M. T., Gaviraghi, L. C., Kirsten, V. R., Kinalski, S. D. S., Hildebrandt, L. M., et al. (2020). Predicting dimensions of clinical-functional conditions and cognition in the elderly. Rev Bras Enferm. 73 Suppl 3, e20190162. doi: 10.1590/0034-7167-2019-0162

44. Solfrizzi, V., Scafato, E., Seripa, D., Lozupone, M., Imbimbo, B. P., D'amato, A., et al. (2017). Reversible Cognitive Frailty, Dementia, and All-Cause Mortality. The Italian Longitudinal Study on Aging. J. Am. Med. Dir. Assoc. 18, 89 e1-89 e8. doi: 10.1016/j.jamda.2016.10.012

45. St John, P. D., Tyas, S. L., Griffith, L. E. and Menec, V. (2017). The cumulative effect of frailty and cognition on mortality - results of a prospective cohort study. Int. Psychogeriatr. 29, 535-543. doi: 10.1017/S1041610216002088

46. Thein, F. S., Li, Y., Nyunt, M. S. Z., Gao, Q., Wee, S. L. and Ng, T. P. (2018). Physical frailty and cognitive impairment is associated with diabetes and adversely impact functional status and mortality. Postgrad. Med. 130, 561-567. doi: 10.1080/00325481.2018.1491779

47. Tsutsumimoto, K., Doi, T., Nakakubo, S., Kim, M., Kurita, S., Ishii, H., et al. (2020). Cognitive Frailty as a Risk Factor for Incident Disability During Late Life: A 24-Month Follow-Up Longitudinal Study. J. Nutr. Health. Aging. 24, 494-499. doi: 10.1007/s12603-020-1365-9

48. Wanaratna, K., Muangpaisan, W., Kuptniratsaikul, V., Chalermsri, C. and Nuttamonwarakul, A. (2019). Prevalence and Factors Associated with Frailty and Cognitive Frailty Among Community-Dwelling Elderly with Knee Osteoarthritis. J. Community Health. 44, 587-595. doi: 10.1007/s10900-018-00614-5

49. Wang, J., Kong, D., Yu, F., Conwell, Y. and Dong, X. (2021). Cognitive deficit, physical frailty, hospitalization and emergency department visits in later life. Aging Ment Health. 25, 521-527. doi: 10.1080/13607863.2019.1699015

50. Xie, B., Ma, C., Chen, Y. and Wang, J. (2021). Prevalence and risk factors of the co-occurrence of physical frailty and cognitive impairment in Chinese community-dwelling older adults. 29, 294-303. doi: 10.1111/hsc.13092

51. Yu, R., Morley, J. E., Kwok, T., Leung, J., Cheung, O. and Woo, J. (2018). The Effects of Combinations of Cognitive Impairment and Pre-frailty on Adverse Outcomes from a Prospective Community-Based Cohort Study of Older Chinese People. Front Med (Lausanne). 5, 50. doi: 10.3389/fmed.2018.00050
