# Supplementary figures and images for: Prevalence and Associated Risk Factors of Cognitive Frailty: A Systematic Review and Meta-Analysis
Source: Front Aging Neurosci. 2022 Jan 28;13:755926. doi: 10.3389/fnagi.2021.755926 (PMC8832102; doi:10.3389/fnagi.2021.755926)

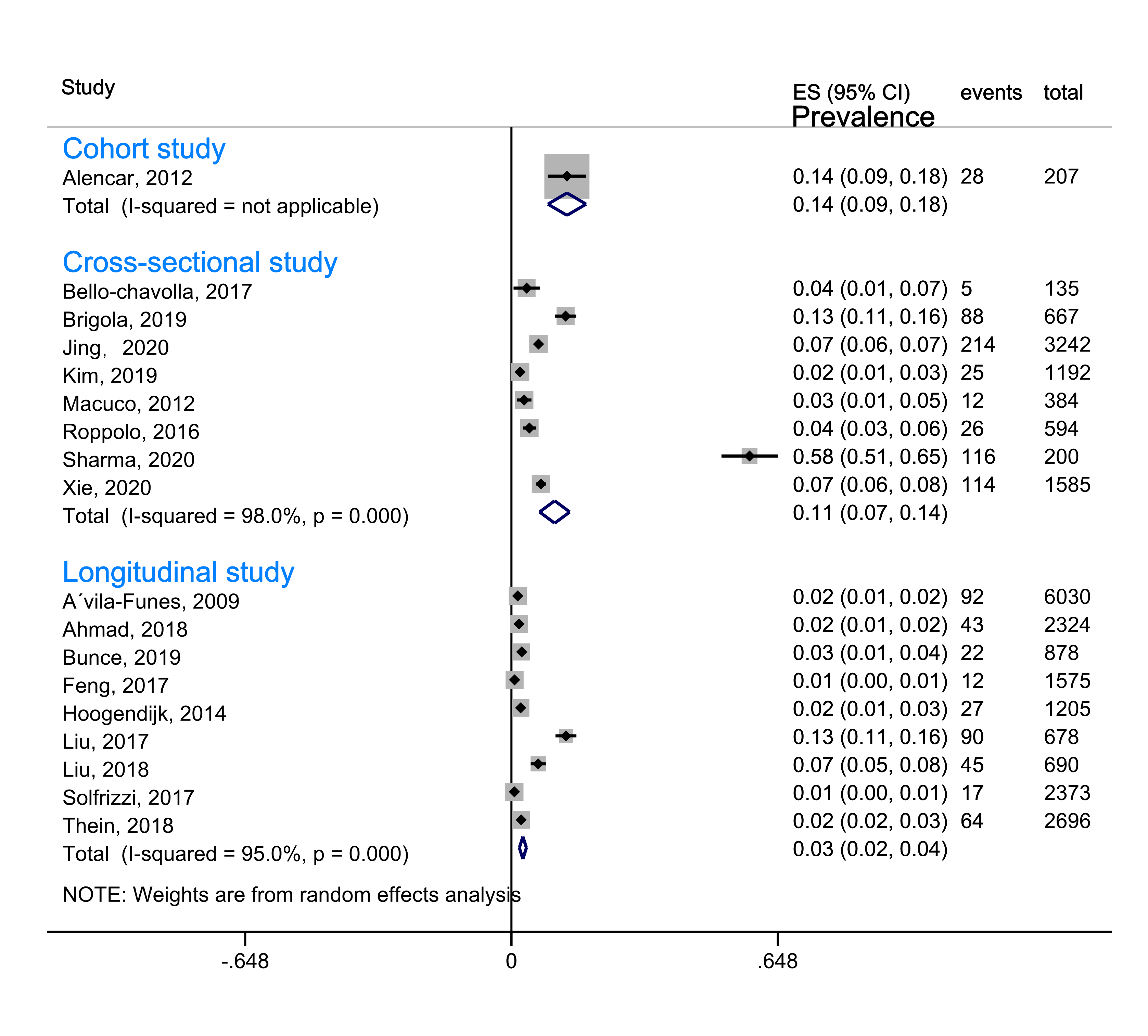

Supplement: Supplementary file 2 [file Image_1.TIFF]
